# Association between Lifestyle Factors and Weight Gain among University Students in Japan during COVID-19 Mild Lockdown: A Quantitative Study

**DOI:** 10.3390/healthcare11192630

**Published:** 2023-09-27

**Authors:** Haruka Arimori, Norio Abiru, Shimpei Morimoto, Tomoya Nishino, Atsushi Kawakami, Akie Kamada, Masakazu Kobayashi

**Affiliations:** 1 Department of Endocrinology and Metabolism, Nagasaki University Hospital, 1-7-1 Sakamoto, Nagasaki 852-8501, Japan; jj20230362@ms.nagasaki-u.ac.jp (H.A.); abirun@nagasaki-u.ac.jp (N.A.); atsushik@nagasaki-u.ac.jp (A.K.); masakazu-f328@nagasaki-u.ac.jp (M.K.); 2 Department of Endocrinology and Metabolism, Division of Advanced Preventive Medical Sciences, Graduate School of Biomedical Sciences, Nagasaki University, 1-7-1 Sakamoto, Nagasaki 852-8501, Japan; 3 Innovation Platform & Office for Precision Medicine, Nagasaki University Graduate School of Biomedical Sciences, 1-7-1 Sakamoto, Nagasaki 852-8501, Japan; mrmt.shmp@gmail.com; 4 Department of Nephrology, Nagasaki University Hospital, 1-7-1 Sakamoto, Nagasaki 852-8501, Japan; tnishino@nagasaki-u.ac.jp; 5 Health Center, Nagasaki University, Nagasaki 852-8521, Japan

**Keywords:** weight gain, lifestyle, young adult, Japan, COVID-19

## Abstract

We aimed to investigate the lifestyle factors influencing weight gain among university students in Japan during the mild lockdown imposed due to the novel coronavirus disease pandemic. In this cross-sectional study, we conducted a questionnaire survey of students who underwent health examinations at Nagasaki University in 2021. Students reporting a weight gain of ≥3 kg were included in the weight gain group; the remaining students were included in the non-weight-gain group. Fisher’s exact test and binary logistic regression were performed to determine the association between weight gain and each lifestyle factor. We included 3059 respondents (response rate: 45.7%), and 9.5% of them reported a weight gain of ≥3 kg. The following factors were associated with weight gain (odds ratio (95% confidence interval), *p* value based on Fisher’s exact test): dining out for four times or more/week (2.16 (1.40, 3.32), *p* = 8.7 × 10^−4^) and gaming time of ≥4 h/day (2.26 (1.45, 3.47), *p* = 2.4 × 10^−4^). Binary logistic regression among the four highest odds ratios revealed that after adjusting for other factors, frequent dining out and prolonged gaming time were significantly associated with weight gain in students during the mild lockdown.

## 1. Introduction

The rapid increase in the prevalence of obesity among young adults is a global health issue [1]. According to a report by the Ministry of Health, Labour and Welfare of Japan, the prevalence of obesity among men in their 20s, 30s, and 40s was 23.1%, 29.4%, and 39.7%, respectively, in 2019 [2]. Kobayashi et al. conducted a cohort study involving university students and reported that metabolic syndrome was observed in 3.3% of men, but not in women [3]. These findings indicate the urgent need for developing countermeasures against obesity in young adults.

The novel coronavirus disease 2019 (COVID-19) outbreak caused a state of emergency in Japan on 7 April 2020. Accordingly, the Japanese government requested individuals to refrain from nonessential and nonurgent outings, avoid social contact to the maximum extent, and stay at home to ensure social distancing, thus preventing the spread of infection. This nonenforceable and nonpunitive request to stay indoors is hereafter referred to as “mild lockdown” in the current study. Although this restriction of activity was considered effective for controlling the spread of COVID-19 infection, it was accompanied by numerous adverse effects, such as lack of exercise, weight gain, behavioral addiction, lack of sun exposure, and social isolation. In particular, staying at home for several months led to changes in social habits, leading to a risk of personal health changes [4,5].

A positive energy balance (in which energy intake exceeds energy expenditure) results in weight gain, and the energy balance is associated with lifestyle such as eating and physical activity [6]. “Lifestyle” comprises daily habits, including diet and exercise. Young adults, particularly university students, experienced major lifestyle changes, such as learning at home and attending online lectures. Further, the voluntary restriction of social club activities and part-time work led to several lifestyle changes in terms of physical activity, diet, and sleep habits, which might be associated with weight gain.

The mild lockdown extended from April 2020 until April 2023, and prolonged lifestyle changes may exacerbate weight gain and health issues among university students. Although young adulthood, such as the university years, is considered a crucial time for developing metabolic abnormalities and increased obesity [7], the impact of lifestyle changes during the mild lockdown on student health has not been adequately examined.

In this study, we investigated the association between weight gain and lifestyle changes caused by 1–2 years of the mild lockdown in students at Nagasaki University, and explored the lifestyle factors and their cutoffs associated with weight gain.

Considering that the study participants were young adults and the survey was conducted during the mild lockdown, the following items were evaluated by questionnaire as lifestyle factors of particular interest: frequency of breakfast, frequency of dining out, drinking habit, alcohol consumption amount, smoking habit, frequency of social club activities, frequency of part-time work, time spent at home, bedtime, wake-up time, sleep duration, gaming time, and internet surfing time.

## 2. Materials and Methods

### 2.1. Participants

The participants were students of Nagasaki University who underwent health examinations in 2020 and 2021. Individuals who understood Japanese, irrespective of their nationality, and provided informed consent were included in this study.

In 2020, among 9179 students at Nagasaki University, 6065 underwent health examinations. Among them, 3722 students completed a questionnaire (response rate, 61.2%). We excluded 1 student with an incomplete questionnaire; therefore, the analysis finally included 3721 students (1850 men and 1871 women).

**Figure 1:**
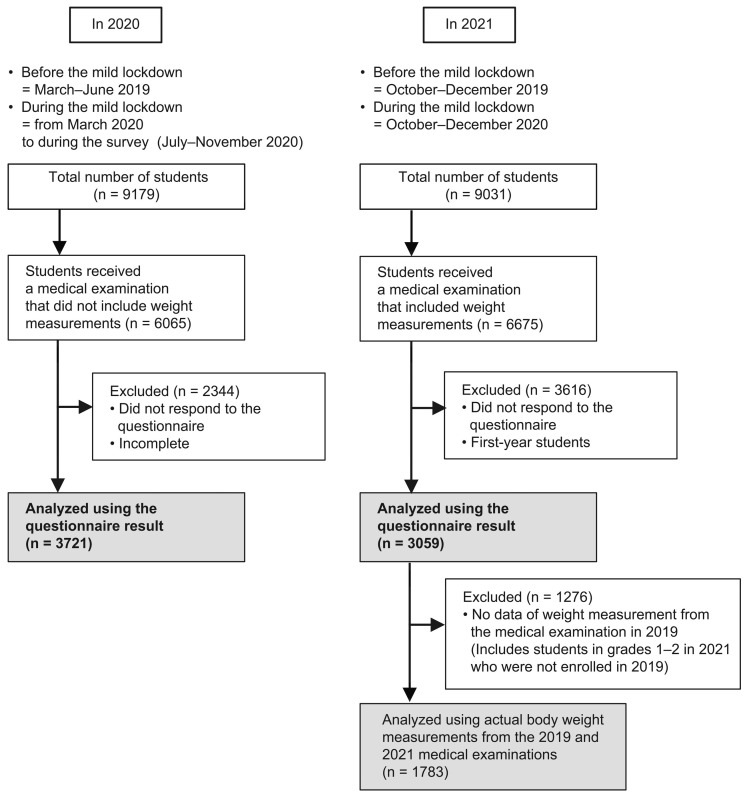
Study flow chart. We defined the before and during mild lockdown periods in our questionnaire as follows: (1) In 2020, before lockdown—March–June 2019, during lockdown—from March 2020 to during the survey (July–November 2020); (2) in 2021, before lockdown—October–December 2019, during lockdown—October–December 2020.

In 2021, among 9031 students at Nagasaki University, 6675 underwent health examinations. Among them, 3059 students (1705 men and 1354 women) who were not first-year students, who consented to the study, and who completed the questionnaire (response rate, 45.7%) were finally included in the analysis (Figure 1).

### 2.2. Study Design

This study was approved by the ethical review board of Nagasaki University (approval number: 20062604) and was conducted in accordance with the principles of the Declaration of Helsinki.

The study was based on a cross-sectional single-center survey conducted from 27 July to 27 November 2020, and from 20 April to 11 June 2021. Researchers and research assistants visited Nagasaki University and requested voluntary participation from students to undergo health examinations in 2020 and 2021. Before implementing the study, written informed consent was obtained from eligible individuals. After obtaining consent in Japanese, the students were requested to complete an electronic questionnaire.

The online survey was distributed as an electronic questionnaire via Google Forms^®^ (QR code provided), which is a social media service. Researchers and research assistants adopted infection prevention measures, such as handwashing and physical distancing, and used surgical masks, face shields, medical gloves, and sanitizing wipes. The questionnaire survey was conducted during waiting periods before and after health examinations. The time required to complete the questionnaire was approximately 5 min. Data were confidentially analyzed by the researchers.

To assess the lifestyle of university students, we developed a novel questionnaire based on the Pittsburgh Sleep Quality Index [8] and National Health and Nutrition Survey [2] of the Ministry of Health and Welfare. Based on the Pittsburgh Sleep Quality Index, we determined bedtime, wake-up time, and sleep duration. Further, we obtained the frequency of breakfast and frequency of dining out from the National Health and Nutrition Survey. The questionnaire included 17 items under the following 6 sections:

1. Personal information obtained via two questions (age and sex);
2. Subjective evaluation of changes in weight and general lifestyle obtained via two questions (weight change—“gain of ≥3 kg,” “gain of <3 kg,” “unchanged,” “loss of <3 kg,” or “loss of ≥3 kg” and general lifestyle change—“changed greatly,” “changed a little,” or “unchanged”);
3. Information regarding physical activity obtained via three questions (time spent at home (h/day), frequency of part-time work (times/week), and frequency of social club activities (times/week));
4. Information regarding diet obtained via two questions (frequency of breakfast (times/week) and frequency of dining out (times/week));
5. Information regarding daily rhythm obtained via three questions (bedtime, wake-up time, and sleep duration (h/day));
6. Information regarding lifestyle obtained via five questions (smoking habit, drinking habit (times/month), alcohol consumption amount (units/time), gaming time (h/day), and internet surfing time (h/day));

We focused on the subjective weight change obtained from the questionnaire, and we included students who reported a weight gain of ≥3 kg in the weight gain (WG) group and the remaining students in the non-WG group. For each category of lifestyle factors in Sections 3–6, categorical variables before and during the mild lockdown were stratified into 3–4 groups. Further, the changes were dichotomized to evaluate whether the lifestyle factor in a patient crossed a threshold during the mild lockdown compared with that before the mild lockdown.

Section 2 included questions regarding changes in weight and lifestyle before and during the mild lockdown in 2020 and 2021. In the evaluation year 2020, the periods of before and after mild lockdown were March–June 2019 and from March 2020 to July–November 2020 (during survey), respectively. In the evaluation year 2021, the periods of before and after mild lockdown were October–December 2019 and October–December 2020, respectively. For each lifestyle factor in Sections 3–6, we evaluated the daily lifestyle of individuals before and during the mild lockdown.

The respondents of the 2021 questionnaire, for whom data regarding actual body weight measurements were obtained from 2019 and 2021, were stratified into five groups based on the amount of change in body weight measurements (ΔBW, kg); we examined the reliability of ΔBW against the questionnaire responses (1—“loss of ≥3 kg” to 5—“gain of ≥3 kg”). ΔBW was stratified according to two versions of the criteria as follows: (1) 1—ΔBW < −3, 2—−3 ≤ ΔBW < −1, 3—−1 ≤ ΔBW < +1, 4—+1 ≤ ΔBW < +3, and 5—ΔBW ≥ +3; and (2) 1—ΔBW < −4.5, 2—−4.5 ≤ ΔBW < 1.5, 3—−1.5 ≤ ΔBW < +1.5, 4—+1.5 ≤ ΔBW < +4.5, and 5—ΔBW ≥ +4.5. Furthermore, the sensitivity and specificity of the questionnaire responses and ΔBW in the five groups were calculated, and the median ΔBW (25th–75th percentile) of each questionnaire response was determined.

### 2.3. Statistical Analysis

#### 2.3.1. Participant Characteristics

Continuous variables other than age were expressed as mean values and standard deviations. Age was presented as mean values and the 25th and 75th percentiles.

The reliability of the weight gain data from the 2021 questionnaire obtained as a substitution for actual body weight measurements in 2019 and 2021 was evaluated as an agreement between discretized body weight measurements using Cronbach’s α coefficient.

#### 2.3.2. Clustering Analysis of the Associations between Changing Lifestyle Factors during the Mild Lockdown

The distance between lifestyle factors was defined as the Cramer’s V statistic calculated from the observed data. Clustering analysis of the associations between the factors was conducted using the Ward’s hierarchical agglomerative algorithm based on the distance.

#### 2.3.3. Association between Weight Gain of ≥3 kg and Each Lifestyle Factor during the Mild Lockdown Using Fisher’s Exact Test and Multivariate Analysis

In categorical data analyses, the healthiest group or the group containing the most individuals was regarded as the reference group. The association between weight gain and lifestyle factors was evaluated using odds ratio (OR) and 95% confidence interval (CI) via Fisher’s exact tests. We conducted a binomial logistic regression analysis wherein the logistic of the weight gain was regressed onto the four independent variables with the highest OR based on Fisher’s exact test. We calculated the *p* value, OR, and 95% CI.

#### 2.3.4. Association between Changes in Lifestyle Factors and Weight Gain of ≥3 kg from before to during the Mild Lockdown Using Empirical Cumulative Distribution Function (ECDF)

The association of changes in lifestyle factors mentioned in Sections 3–6 with weight gain was evaluated as the ratio of the McNamar’s chi-square statistic, wherein the numerator was the chi-square statistic on the WG group, whereas the denominator was the statistic on all students. Each chi-square statistic was obtained from the contingency table of dichotomized changes in lifestyle factors. The null distribution of the ratio for each factor was obtained as ECDF with permutations of the labels (Yes/No) of “weight gain”. For each factor, as ECDF returned the extreme value for the observed data, a cutoff threshold was determined between the adjacent categories.

Statistical analyses were conducted using R version 4.0.3, developed by the R Development Core Team [9].

## 3. Results

### 3.1. Participant Characteristics

Responses were obtained from 3721 students (mean age, 21.3 years) in 2020 and 3059 students (mean age, 21.8 years) in 2021. The WG group consisted of 224 (6.0%) and 290 (9.5%) students in 2020 and 2021, respectively; the number of students in the WG group was significantly higher in 2021 (*p* < 0.01) than in 2020. In both years, the WG group comprised a higher number of male students than the non-WG group; further, the body height, body weight, and body mass index of students were higher in the WG group (*p* < 0.05). In both years, the proportion of students in the WG group who experienced a major change in their overall lifestyle was higher than that in the non-WG group (*p* < 0.01, Table 1).

Regarding the 1783 students who participated in the 2021 questionnaire and for whom actual body weight measurements were obtained during health examinations in 2019 and 2021 (i.e., students who gained not only subjective weight change based on the questionnaire but also objective actual weight change based on health examinations), we evaluated the extent of changes in questionnaire responses compared with actual body weight measurements (Figure S1). The sensitivity and specificity values for a weight gain of ≥3 kg and ΔBW of ≥4.5 kg were 0.47 and 0.95, whereas those for ΔBW of ≥3 kg were 0.37 and 0.97, respectively. The median ΔBW was +4.6 (+2.9 to +6.9) kg in the WG group and −0.5 (−2.4 to +1.6) kg in the non-WG group. The confusion matrix and Cronbach’s α coefficients are shown in Table S1.

### 3.2. Clustering Analysis of the Associations between Changing Lifestyle Factors during the Mild Lockdown

**Figure 2:**
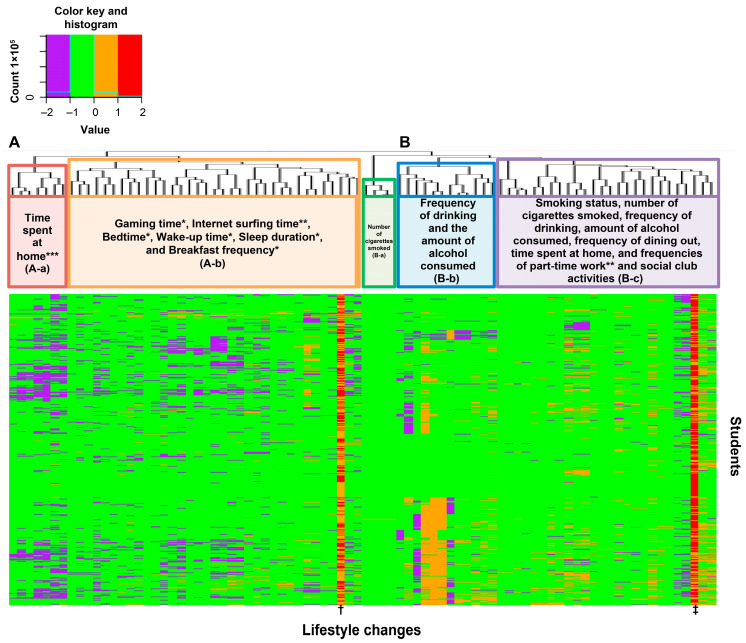
Heatmap showing the clustering of lifestyle changes before and during the mild lockdown. The vertical axis represents students, while the horizontal axis represents each cutoff for lifestyle changes. Purple indicates a change from above to below the cutoff. Light green indicates no change (i.e., above to above or below to below the cutoff). Orange indicates a change from below to above the cutoff. *: Lifestyle factors found to be associated with weight gain using Fisher’s exact test across all students; ** and *** indicate lifestyle factors found to be associated with weight gain using Fisher’s exact test across male students and female students, respectively. we observed two major groups (A and B) of change in lifestyle factors as well as two and three subgroups of factors under groups (**A**) (A-a–A-b) and (**B**) (B-a–B-c), respectively. †: sex. ‡: absence of dramatic lifestyles changes. Regarding sex, orange represents male students and red represents female students. For overall lifestyle change, orange indicates dramatic change and red indicates no dramatic change.

To identify the categories of lifestyle factors in which the changes during the mild lockdown for all participants (irrespective of body weight change) in 2021 were mutually dependent, we conducted clustering analyses (Figure 2). Because we used Cramer’s V statistic as a measure of the distance between the factors, nonlinear and non-rank-ordering associations were included in our findings. The closer the items are to each other, the more strongly related the factors of change in lifestyle factors, as presented in the tree diagram in Figure 2. Changes in lifestyle factors that were indicated to be related were grouped based on this tree diagram. Consequently, we observed two major groups (A and B) of change in lifestyle factors as well as two and three subgroups of factors under groups A (A-a–A-b) and B (B-a–B-c), respectively. Group A included “changes in the time spent at home” (red) and “changes in gaming time,” “sleeping rhythm,” and “frequency of breakfast” (orange). Group B included “changes in smoking habit” (green), “changes in alcohol consumption amount” (blue), and other changes (such as changes in the frequencies of part-time job, social club activities, dining out, drinking, and smoking) (purple) (Figure 2).

### 3.3. Association between Weight Gain of ≥3 kg and Each Lifestyle Factor during the Mild Lockdown Using Fisher’s Exact Test

**Figure 3:**
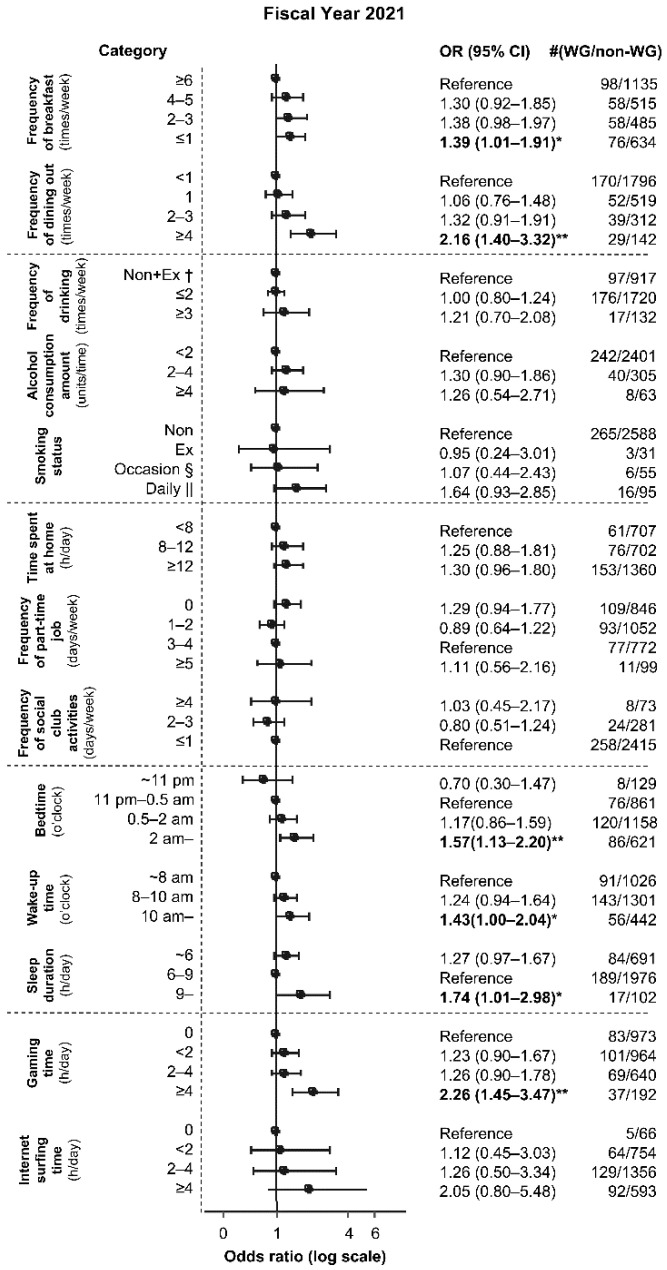
Relationship between weight gain of ≥3 kg and each lifestyle factor during the mild lockdown in all students. * *p* < 0.05; ** *p* < 0.01. † Non: nondrinker or nonsmoker, Ex: ex-drinker or ex-smoker. § Occasion: occasional smoker. || Daily: daily smoker. OR: odds ratio. WG: weight gain group (students who responded that they experienced a weight gain of ≥3 kg in the 2021 survey about weight change from before to during the mild lockdown). Non-WG: non-weight-gain group (students who were excluded in the WG group from the total, i.e., those who responded that they experienced a weight gain of <3 kg, no weight change, weight loss of <3 kg, or weight loss of ≥3 kg in the 2021 survey about weight change from before to during the mild lockdown). Error bars indicate 95% confidence intervals.

The following factors were found to be associated with a weight gain of ≥3 kg (OR (95% CI), *p* value based on Fisher’s exact test of the 2021 questionnaire data): frequency of breakfast of ≤1 time/week (1.39 (1.01, 1.91), *p* = 4.7 × 10^−2^), frequency of dining out of four or more times/week (2.16 (1.40, 3.32), *p* = 8.7 × 10^−4^), bedtime at or after 2 am (1.57 (1.13, 2.20), *p* = 7.4 × 10^−3^), wake-up time at or after 10 am (1.43 (1.00, 2.04), *p* = 4.9 × 10^−2^), sleep duration of ≥9 h (1.74 (1.01, 2.98), *p* = 4.8 × 10^−2^), and gaming time of ≥4 h/day (2.26 (1.45, 3.47), *p* = 2.4 × 10^−4^) (Figure 3).

**Figure 4:**
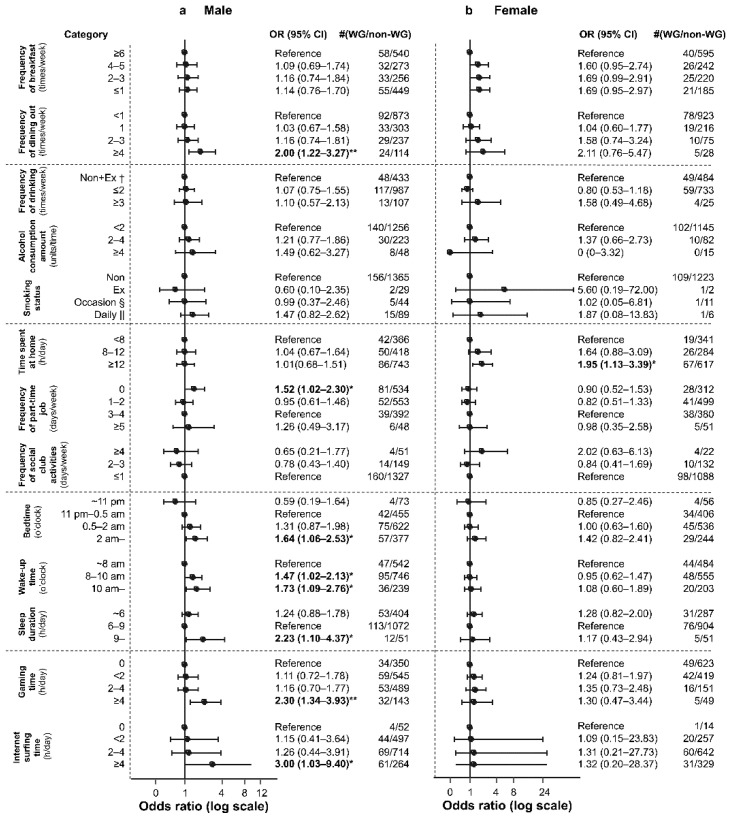
Relationship between weight gain of ≥3 kg and each lifestyle factor during the mild lockdown in (**a**) male students and (**b**) female students. * *p* < 0.05; ** *p* < 0.01. † Non: nondrinker or nonsmoker, Ex: ex-drinker or ex-smoker. § Occasion: occasional smoker. || Daily: daily smoker. OR: odds ratio. WG: weight gain group (students who responded that they experienced a weight gain of ≥3 kg in the 2021 survey about weight change from before to during the mild lockdown). Non-WG: non-weight-gain group (students who were excluded in the WG group from the total, i.e., those who responded that they experienced a weight gain of <3 kg, no weight change, weight loss of <3 kg, or weight loss of ≥3 kg in the 2021 survey about weight change from before to during the mild lockdown). Error bars indicate 95% confidence intervals.

Furthermore, in the analysis based on sex, the following factors were found to be associated with a weight gain of ≥3 kg in male students: frequency of dining out of ≥4 times/week (2.00 (1.22, 3.27), *p* = 7.4 × 10^−3^), part-time work frequency of 0 times/week (1.52 (1.02, 2.30), *p* = 4.8 × 10^−2^), bedtime at or after 2 am (1.64 (1.06, 2.53), *p* = 2.5 × 10^−2^), wake-up time between 8 and 10 am (1.47 (1.02, 2.13), *p* = 3.9 × 10^−2^), wake-up time at or after 10 am (1.73 (1.09, 2.76), *p* = 2.5 × 10^−2^), sleep duration of ≥9 h (2.23 (1.10, 4.37), *p* = 2.8 × 10^−2^), gaming time of ≥4 h/day (2.30 (1.34, 3.93), *p* = 1.8 × 10^−3^), and internet surfing time of ≥4 h/day (3.00 (1.03, 9.40), *p* = 3.4 × 10^−2^) (Figure 4a). In female students, only one factor was associated with a weight gain of ≥3 kg: time spent at home of ≥12 h/day (1.95 (1.13, 3.39), *p* = 1.3 × 10^−2^) (Figure 4b).

Overall, we found that in 2021, weight gain in students was associated with skipping breakfast, frequent dining out, delayed sleep–wake phase, and gaming time. In male students, weight gain was associated with frequent dining out, part-time job frequency, delayed sleep–wake phase, gaming time, and internet surfing time, whereas in female students, it was associated with time spent at home.

### 3.4. Multivariate Analysis

We conducted binary logistic regression of the four factors with the highest OR based on Fisher’s exact test. These top four factors (OR; 95% CI) included frequency of dining out of four or more times/week (2.16; (1.40, 3.32)), sleep duration of ≥9 h (1.74; (1.01, 2.98)), gaming time of ≥4 h (2.26; (1.45, 3.47)), and internet surfing time of ≥4 h (2.05; (0.80, 5.48)) (Figure 3). After adjusting for the other three factors, weight gain was still found to be associated with frequency of dining out of four or more times/week (log OR, 0.72–0.75; *p* = 6.1 × 10^−4^ to 1.0 × 10^−3^) and gaming time of ≥4 h (log OR, 0.67–0.77; *p* = 3.5 × 10^−4^ to 2.3 × 10^−3^) (Table 2).

Regarding sex differences, the top four factors (OR; 95% CI) identified from Fisher’s exact test for male students included frequency of dining out of four or more times/week (2.00; (1.22, 3.27)), sleep duration of ≥9 h (2.23; (1.10, 4.37)), gaming time of ≥4 h (2.30; (1.34, 3.93)), and internet surfing time of ≥4 h (3.00; (1.03, 9.40)) (Figure 4a). After adjusting for the other three factors, weight gain was still found to be associated with frequency of dining out of four or more times/week (log OR, 0.62–0.68; *p* = 6.8 × 10^−3^ to 1.4 × 10^−2^) and gaming time of ≥4 h (log OR, 0.78–0.82; *p* = 2.0 × 10^−3^ to 3.9 × 10^−3^). However, after adjusting for internet surfing time, no association between weight gain and gaming time was observed (log OR, 0.52; *p* = 6.0 × 10^−2^). Incidentally, sleep duration of ≥9 h was found to be associated with weight gain only after adjusting for the frequency of dining out (log OR, 0.78; *p* = 2.0 × 10^−2^), and internet surfing time of ≥4 h was found to be associated with weight gain only after adjusting for sleep duration (log OR, 1.09; *p* = 4.0 × 10^−2^; Table S2).

The top four factors (OR; 95% CI) identified from the Fisher’s exact test for female students included frequency of dining out of four or more times/week (2.11; (0.76, 5.47)), occasional smoking (5.60; (0.19, 72.00)), time spent at home of ≥12 h (1.95; (1.13, 3.39)), and frequency of social club activities of four or more times/week (2.02; (0.63, 6.13)) (Figure 4b). After adjusting for the other three factors, weight gain was still found to be associated with time spent at home of ≥12 h (log OR, 0.67–0.68; *p* = 1.00 × 10^−2^) (Table S3). Furthermore, the factor with a high log OR of >0.6 and *p* value of ≥0.05 in all students was internet surfing time of ≥4 h (adjusted for frequency of dining out and sleep duration). Moreover, factors with a high log OR of >0.6 and *p* value of ≥0.05 in male students included internet surfing time of ≥4 h (adjusted for frequency of dining out and gaming time) and sleep duration of ≥9 h (adjusted for gaming time and internet surfing time), whereas those in female students included frequency of dining out of four or more times/week and occasional smoking (adjusted for the other three factors) (Table 2, Tables S2 and S3).

### 3.5. Association between Lifestyle Changes and Weight Gain of ≥3 kg from before to during the Mild Lockdown Using ECDF

We examined the association of lifestyle changes with the weight gain from before the mild lockdown to the time of survey; this association has not been analyzed in studies to date. We extracted the ECDF value of changes in each cutoff value for lifestyle factors in 2021 as well as for weight gain of ≥3 kg based on the ratio of the McNemar chi-square statistics (the strength of lifestyle change before and during the mild lockdown) obtained from all participants to that (the strength of lifestyle change before and during the mild lockdown) obtained from the WG group. Lifestyle changes with an ECDF value of close to 1 were considered to be highly associated with a weight gain of ≥3 kg. For each lifestyle factor, we extracted the cutoff for the change with the greatest ECDF and the associated ECDF; these rankings are presented in Table 3a. The higher the ECDF value (close to 1), the higher the ranking, indicating that lifestyle changes are highly associated with a weight gain of ≥3 kg.

Factors with a high-ranking ECDF value included bedtime at or after 2 am, prolonged internet surfing time of >0 h, prolonged time spent at home of ≥16 h, prolonged sleep duration of ≥9 h, and prolonged gaming time of ≥1 h/day (ECDF ≥ 0.96). In terms of sex, although the cutoff value differed between sexes, the maximum ECDF value of changes in these lifestyle factors was high (ECDF ≥ 0.92) (Table 3b,c).

## 4. Discussion

Imposing lockdown during the COVID-19 pandemic was an effective measure for preventing the spread of the infection; however, staying at home for a long period can threaten personal health and cause harmful effects such as weight gain [4,10].

The study revealed that the WG group, based on self-reports in questionnaires, increased significantly from 2020 to 2021, and that the WG group demonstrated significantly higher body mass index (BMI) values obtained from actual height and weight measurements from student health examinations than the non-WG group. This indicates that prolonged COVID-19 infection was associated with actual weight gain among the students. Additionally, the WG group consisted of more boys than the non-WG group, and the majority of them experienced major changes in their overall lives. Several studies have reported weight gain during the COVID-19 lockdown [11,12]. On the other hand, no consistent results have been reported on the association between weight gain and sex differences. Some studies reported no sex differences [13,14], others reported significantly greater weight gain in women [11,12,15,16,17], and still others reported significantly greater weight gain in obese adolescents and children than in men [18,19]. The variation in these results may be due to differences in age, race, education level, and other factors.

Clustering analysis of changes in lifestyle factors for all students revealed that “change in time spent at home,” “change in gaming time,” “change in sleep rhythm,” and “change in frequency of breakfast” were close and associated with each other, indicating that gaming time is associated with daily lifestyle factors among young people. Associations between pathological game use and skipping breakfast, late bedtime, few friends, and lack of parent–child interaction have been reported in Japanese elementary school students [20]. The present study of university students revealed similar associations between gaming time, skipping breakfast, and late bedtime. Our study did not include friendship and parent–child interaction in the survey item. However, our study revealed a decrease in the frequency of social club activities and a decrease in drinking frequency were observed before and during the COVID-19 mild lockdown (the percentages of students with the frequency of social club activities of more than two times/week in students who undertook social club activities—before 62.6% and during 28.8%; the percentages of students who drank with someone more than once/month—before 39.9% and during 14.8%), and a decrease in socializing with friends may be an important item in future surveys.

The results of the 2021 Fisher test and binomial logistic regression analysis revealed that the frequency of dining out of more than four times/week and gaming times of >4 h/day for all and male students, and time spent at home of >12 h/day for female students, were significantly associated with weight gain. The analysis using ECDF, which focused on changes before and during the COVID-19 mild lockdown, revealed that weight gain was associated with longer gaming time and longer time spent at home, similar to the above, but was less associated with increased frequency of dining out. This indicates that the association between increased frequency of dining out and weight gain did not appear during the process of change before and during the mild COVID-19 lockdown, but rather that it may have already been latent and became apparent with the spread of COVID-19 infection.

Food during dining out is reported to be high in calories, sodium, saturated fatty acids, and carbohydrates [21], and low in terms of the American Heart Association (AHA) diet score, which has been validated as a risk factor for cardiovascular disease [22]. A study of Japanese adults in 2015, before the spread of COVID-19 infection, reported that individuals with obesity accounted for 40.0% of the population who dined out at least twice a day and 22.7–28.9% of the population who dined out less than twice a day [23]. A Korean study reported that dining out habits increased the risk of weight gain during the COVID-19 pandemic [24]. However, the percentage of students in our study who had the habit of dining out daily was 2.7% before compared to 1.8% during the mild COVID-19 lockdown, indicating a lower frequency of dining out during the COVID-19 pandemic due to concerns about the risk of infection [25]. Thus, young people who dine out frequently are a small group of students, but may be a high-risk population for weight gain, regardless of the COVID-19 pandemic. A need to raise awareness among young people is considered because COVID-19 infection is expected to increase with the frequency of dining out as the COVID-19 pandemic converges in the future.

The present study confirmed the association between gaming time and weight gain for all students and male students. Gaming and internet addiction disorders are concepts that have long been established; however, the recent increase in the prevalence of gaming disorder, particularly among young adults, has drawn attention as a health issue. In 2019, gaming disorder was proposed as an important new disease concept to be adopted into the World Health Organization Eleventh Revision of the International Classification of Diseases [26,27]. The prevalence of gaming disorder among young adults is reported to be 10–15% in Asia and 1–10% in the Western countries [28]. Additionally, studies evaluating the health status of adolescents and young adults during the COVID-19 pandemic reported that internet addiction and gaming disorders exert an adverse effect on health, causing disorders, such as obesity and weight gain, due to sleep deficit [29,30,31]. Moreover, in Japan, with the spread of COVID-19 infection, the incidence of internet gaming disorder has increased [32], and the association with weight gain may have been confirmed as one of the phenotypes of this health disorder in our study. Reportedly, prolonged gaming and internet surfing times can cause weight gain owing to factors such as a higher number of meals consumed during screen time and increased exposure, preference, and desire to purchase high-calorie foods [33]. Sedentary behavior is known to be consciously associated with obesity [34], thus increased sedentary behavior due to gaming may lead to weight gain. It has also been reported that internet addiction and gaming disorders are highly associated with mental health issues, such as depression, anxiety [35], eating disorders [36], and sleep disturbance [37,38,39,40,41,42]. In particular, sleep disturbance may disrupt the circadian rhythm and is associated with obesity and hepatic and intestinal diseases caused by the dysregulation of the physiological functions of hepatocytes, gastrointestinal cells, and adipocytes [43,44].

The current study indicated that time spent at home is associated with weight gain in female students. Ming et al. reported that women and non-obese men had weight gain, but men with a BMI of >24 kg/m^2^ demonstrated weight loss during semi-lockdown (27 January 2020, to 1 March 2020, the period during which the Chinese government imposed restrictions on dining out, prohibiting going out, and quarantining). This is because men with a BMI of >24 kg/m^2^ originally had a high awareness of weight management, but due to work, they socialized and drank a lot, resulting in a high BMI; however, their socialization was reduced due to the impact of semi-lockdown [45]. Pitol et al. reported that outdoor exercise time decreased during lockdown [46]. Our study considered the possibility that Japanese female students, who originally had fewer opportunities for drinking parties than male students, did not lose weight due to fewer drinking parties, but rather experienced weight loss due to prolonged time spent at home before and during the COVID-19 pandemic. Furthermore, young Japanese women have been reported to have less access to places to exercise than men, and many dislike exercise [2], and the possibility that this was related to the present results should also be considered.

This study has several limitations. First, as this was a study based on questionnaire responses, there were no objective data, such as the height and weight of the participants; therefore, biases may have occurred due to self-reporting (recall and social desirability biases) and due to the limitation of the sample population to students who provided consent. Second, this study included students of a single Japanese institution, leading to difficulties in the generalization of the study results. To investigate the causal association of weight gain with factors such as gaming time, a multicenter prospective study is warranted in the future. Third, the IPAQ questionnaire [47] and the 3-day food recall questionnaire [48] are suitable for evaluating the lifestyle habits and dietary composition of young Japanese people, but these were not used in this study. To conduct a more detailed lifestyle assessment in the future, performing additional research using similar questionnaires seems necessary.

## 5. Conclusions

In conclusion, this study investigated lifestyle factors influencing weight gain among university students in a particular environment, i.e., the mild lockdown, and confirmed the association of weight gain with the frequency of dining out, gaming time, and prolonged time spent at home. These are considered important as potential causes of health problems among young people in general, and proactive early intervention and measures to prevent weight gain should be discussed in the future.

## Figures and Tables

**Table 1:** Characteristics of participants from the evaluation years (a) 2020 and (b) 2021.

**(a) Evaluation year 2020**															
	**All**		**Weight gain group**		**Non-weight-gain**		***p* value †**	**Classification of the non-weight-gain group**							
			**(Weight gain of** **≥3 kg)**		**group**			**Weight gain of** **<3 kg**		**No change**		**Weight loss of** **<3 kg**		**Weight loss of** **≥3 kg**	
N (male, %)	3721	(1850, 49.7)	224	(138, 61.6)	3497	(1712, 49.0)	<0.01 **	656	(322, 49.1)	1960	(1069, 54.5)	556	(200, 36.0)	325	(121, 37.2)
Age (25th–75th percentile)	21.3	(19–22)	22.2	(20–23)	21.2	(19–22)	<0.01 **	21.5	(20–22)	21.2	(20–22)	21	(19–22)	20.8	(19–22)
Height (SD), cm ‡	164.3	(±8.6)	166.1	(±8.4)	164.2	(±8.5)	0.01 *	163.7	(±8.7)	165.1	(±8.6)	162.6	(±8.3)	163.2	(±8.1)
Body weight (SD), kg ‡	57.1	(±10.6)	65.8	(±12.9)	56.6	(±10.2)	<0.01 **	59.6	(±11.0)	56.4	(±10.0)	54	(±9.0)	56.4	(±10.3)
Body mass index (SD), kg/cm^2^ ‡	21	(±3.0)	23.8	(±3.9)	20.9	(±2.8)	<0.01 **	22.1	(±8.4)	22.1	(±2.9)	20.3	(±2.5)	21.1	(±2.7)
Drastic changes in lifestyles, n (%)	1051	28.2	101	45.1	950	27.2	<0.01 **	198	30.2	455	23.2	171	30.8	126	38.8
Serious anxiety, n (%)	739	19.9	60	26.8	679	19.4	<0.01 **	150	22.9	306	15.6	149	26.8	74	22.8
**(b) Evaluation year 2021**															
	**All**		**Weight gain group**		**Non-weight-gain**		***p* value †**	**Classification of the non-weight-gain group**							
			**(Weight gain of** **≥3 kg)**		**group**			**Weight gain of** **<3 kg**		**No change**		**Weight loss of** **<3 kg**		**Weight loss of** **≥3 kg**	
N (male, %)	3059	(1705, 55.7)	290	(178, 61.4)	2769	(1527, 55.1)	0.05 *	524	(273, 52.1)	1517	(886, 58.4)	469	(240, 51.2)	259	(128, 49.0)
Age (25th–75th percentile)	21.8	(20–23)	22.1	(20–23)	21.7	(20–22)	0.04 *	22.1	(20–23)	21.6	(20–22)	21.7	(20–22)	21.6	(20–22)
Height (SD), cm ‡	165.6	(±8.5)	166.5	(±8.1)	165.5	(±8.6)	0.04 *	164.8	(±8.6)	165.9	(±8.6)	164.9	(±8.4)	165.3	(±8.6)
Body weight (SD), kg ‡	58.3	(±11.0)	67.1	(±12.2)	57.4	(±10.4)	<0.01 **	60.9	(±10.8)	57.1	(±10.2)	54.7	(±9.7)	56.8	(±10.5)
Body mass index (SD), kg/cm^2^ ‡	21.2	(±3.0)	24.1	(±3.5)	20.9	(±2.8)	<0.01 **	22.3	(±2.8)	20.6	(±2.7)	20	(±2.5)	20.7	(±2.7)
Drastic changes in lifestyle, n (%)	928	30.3	133	45.9	795	28.7	<0.01 **	167	31.9	371	24.5	161	34.3	96	37.1
Serious anxiety, n (%)	550	18	81	27.9	469	16.9	<0.01 **	100	19.1	196	12.9	99	21.1	74	28.6

Body mass index: body weight (kg)/height (cm^2^). Weight gain group (students who reported a weight gain of ≥3 kg in the 2021 survey from before to during the mild lockdown); non-weight-gain group (students who reported a weight gain of <3 kg, no weight change, weight loss of <3 kg, or weight loss of ≥3 kg in the 2021 survey from before to during the mild lockdown). † *p* value: the *p* value based on Fisher’s exact or Welch’s *t* tests in weight gain and non-weight-gain groups. * *p* < 0.05, ** *p* < 0.01. ‡ Anthropometric factors (height, body weight, and body mass index) were obtained from the physical examination data in year 2021. The number of respondents to the 2020 questionnaire survey who provided data on anthropometric factors was 2337 (weight gain group, 129; non-weight-gain group, 2208). The number of respondents to the 2021 questionnaire survey who provided data on anthropometric factors was 3035 (weight gain group, 287; non-weight-gain group, 2746).

**Table 2:** Binary logistic regression analysis of the four lifestyle factors (with the highest OR in the Fisher’s exact test) associated with a weight gain of >3 kg during mild lockdown in all students.

Lifestyle Factors	Category	Units	Estimate of Log Odds Ratio	(95% Confidence Interval)	*p* Value	
Frequency of dining out and sleep duration						
Frequency of dining out	<1	time/week	Reference			
Frequency of dining out	1	time/week	0.06	(−0.27 to 0.38)	0.71	
Frequency of dining out	2–3	times/week	0.28	(−0.10 to 0.63)	0.14	
Frequency of dining out	≥4	times/week	0.75	(0.31 to 1.17)	6.1 × 10^−4^	***
Sleep duration	6–9	h/day	Reference			
Sleep duration	<6	h/day	0.23	(−0.04 to 0.50)	0.10	
Sleep duration	≥9	h/day	0.53	(−0.04 to 1.04)	0.05	
Frequency of dining out and gaming time						
Frequency of dining out	<1	time/week	Reference			
Frequency of dining out	1	time/week	0.04	(−0.30 to 0.36)	0.82	
Frequency of dining out	2–3	times/week	0.23	(−0.15 to 0.59)	0.22	
Frequency of dining out	≥4	times/week	0.72	(0.28 to 1.14)	1.0 × 10^−3^	**
Gaming time	0	h/day	Reference			
Gaming time	<2	h/day	0.2	(−0.11 to 0.50)	0.20	
Gaming time	2–4	h/day	0.2	(−0.13 to 0.54)	0.23	
Gaming time	≥4	h/day	0.77	(0.34 to 1.18)	3.5 × 10^−4^	***
Frequency of dining out and internet surfing time						
Frequency of dining out	<1	time/week	Reference			
Frequency of dining out	1	time/week	0.05	(−0.29 to 0.37)	0.77	
Frequency of dining out	2–3	times/week	0.26	(−0.12 to 0.62)	0.17	
Frequency of dining out	≥4	times/week	0.74	(0.29 to 1.16)	7.5 × 10^−4^	***
Internet surfing time	0	h/day	Reference			
Internet surfing time	<2	h/day	0.09	(−0.76 to 1.17)	0.85	
Internet surfing time	2–4	h/day	0.21	(−0.62 to 1.27)	0.66	
Internet surfing time	≥4	h/day	0.68	(−0.16 to 1.75)	0.15	
Sleep duration and gaming time						
Sleep duration	6–9	h/day	Reference			
Sleep duration	<6	h/day	0.21	(−0.06 to 0.48)	0.12	
Sleep duration	≥9	h/day	0.45	(−0.13 to 0.96)	0.11	
Gaming time	0	h/day	Reference			
Gaming time	<2	h/day	0.21	(−0.09 to 0.52)	0.18	
Gaming time	2–4	h/day	0.23	(−0.11 to 0.56)	0.19	
Gaming time	>4	h/day	0.76	(0.33 to 1.18)	3.9 × 10^−4^	***
Sleep duration and internet surfing time						
Sleep duration	6–9	h/day	Reference			
Sleep duration	<6	h/day	0.24	(−0.04 to 0.51)	0.09	
Sleep duration	≥9	h/day	0.43	(−0.14 to 0.94)	0.12	
Internet surfing time	0	h/day	Reference			
Internet surfing time	<2	h/day	0.12	(−0.74 to 1.19)	0.81	
Internet surfing time	2–4	h/day	0.24	(−0.59 to 1.30)	0.62	
Internet surfing time	≥4	h/day	0.7	(−0.14 to 1.77)	0.14	
Gaming time and internet surfing time						
Gaming time	0	h/day	Reference			
Gaming time	<2	h/day	0.28	(−0.03 to 0.60)	0.08	
Gaming time	2–4	h/day	0.29	(−0.06 to 0.63)	0.10	
Gaming time	≥4	h/day	0.67	(0.23 to 1.09)	2.3 × 10^−3^	**
Internet surfing time	0	h/day	Reference			
Internet surfing time	<2	h/day	−0.08	(−0.96 to 1.01)	0.87	
Internet surfing time	2–4	h/day	0.04	(−0.80 to 1.12)	0.93	
Internet surfing time	≥4	h/day	0.49	(−0.37 to 1.57)	0.31	

** *p* < 0.01; and *** *p* < 0.01. We conducted binary logistic regression analysis of the four factors with the highest OR in the Fisher’s exact test. These factors included frequency of dining out of ≥4 times/week, sleep duration of ≥9 h, gaming time of ≥4 h, and internet surfing time of ≥4 h.

**Table 3:** Ranking of ECDF values for the largest value of each lifestyle change factor in 2021 in (a) all students, (b) male students, and (c) female students.

	(a) All				(b) Male				(c) Female			
Rank	Changes in lifestyle factors		Cutoff	ECDF	Rank		Cutoff	ECDF	Rank		Cutoff	ECDF
1	Bedtime	≥	26 o’clock	0.999	2	≥	26 o’clock	0.988	4	≥	26 o’clock	0.984
2	Internet surfing time	≥	0 h/day	0.998	4	≥	4 h/day	0.982	3	≥	0 h/day	0.987
3	Time spent at home	≥	16 h/day	0.992	6	≥	10 h/day	0.961	1	≥	20 h/day	1
4	Sleep duration	≥	9 h/day	0.971	5	≥	9 h/day	0.981	5	≥	8 h/day	0.955
5	Gaming time	≥	1 h/day	0.97	8	≥	4 h/day	0.924	2	≥	0 h/day	0.998
6	Frequency of smoking	=	Daily	0.95	7	=	Daily	0.948	6	=	Occasionally or daily	0.942
7	Frequency of part-time job	<	1 day/week	0.947	1	<	1 day/week	0.992	13	≥	5 day/week	0.428
8	Wake-up time	≥	12 o’clock	0.946	3	≥	12 o’clock	0.985	11	≥	10 o’clock	0.796
9	Frequency of social club activity	<	5 days/week	0.811	9	<	5 days/week	0.816	10	<	1 days/week	0.816
10	Frequency of dining out	≥	14 times/week	0.751	10	≥	14 times/week	0.763	7	≥	7 times/week	0.875
11	Frequency of breakfast	≥	2 times/week	0.691	13	<	5 times/week	0.502	8	<	5 times/week	0.873
12	Amount of alcohol consumed	<	3 units/time	0.656	11	<	3 units/time	0.743	12	≥	2 units/time	0.518
13	Frequency of drinking	≥	20 times/month	0.604	12	<	12 times/month	0.728	9	≥	20 times/month	0.828

ECDF: empirical cumulative distribution function. ECDF analysis was performed for each category of lifestyle changes before and during the mild lockdown using the ratio of the McNemar chi-square value (the strength of lifestyle change) for all participants to that for the weight gain group as the statistic. Lifestyle changes with an ECDF value of close to 1 were considered to be highly associated with weight change.

## Data Availability

The presented data are available upon request from the corresponding author. The data are not publicly available to prevent the misuse and misinterpretation of data.

## Supplementary Materials

The following supporting information can be downloaded at: https://www.mdpi.com/2227-9032/11/19/2630/s1, Figure S1: Self-reported weight change in the fiscal year 2021 questionnaire and the amount of actual weight change based on student health examination data from fiscal years 2019 to 2021; Table S1: Confusion matrix and Cronbach’s α coefficients of self-reported weight change in the fiscal year 2021 questionnaire and the amount of actual weight change based on student health examination data from fiscal years 2019 to 2021; Table S2: Binary logistic regression analysis for the lifestyle factors associated with a weight gain of >3 kg during mild lockdown of the four factors with the highest OR in the Fisher’s exact test in male students; Table S3. Binary logistic regression analysis for the lifestyle factors associated with a weight gain of >3 kg during mild lockdown of the four factors with the highest OR in the Fisher’s exact test in female students.
