# Correction: Arimori et al. Association between Lifestyle Factors and Weight Gain among University Students in Japan during COVID-19 Mild Lockdown: A Quantitative Study. *Healthcare* 2023, *11*, 2630

**DOI:** 10.3390/healthcare12040433

**Published:** 2024-02-08

**Authors:** Haruka Arimori, Norio Abiru, Shimpei Morimoto, Tomoya Nishino, Atsushi Kawakami, Akie Kamada, Masakazu Kobayashi

**Affiliations:** 1 Department of Endocrinology and Metabolism, Nagasaki University Hospital, 1-7-1 Sakamoto, Nagasaki 852-8501, Japan; jj20230362@ms.nagasaki-u.ac.jp (H.A.); abirun@nagasaki-u.ac.jp (N.A.); atsushik@nagasaki-u.ac.jp (A.K.); masakazu-f328@nagasaki-u.ac.jp (M.K.); 2 Department of Endocrinology and Metabolism, Division of Advanced Preventive Medical Sciences, Graduate School of Biomedical Sciences, Nagasaki University, 1-7-1 Sakamoto, Nagasaki 852-8501, Japan; 3 Innovation Platform & Office for Precision Medicine, Nagasaki University Graduate School of Biomedical Sciences, 1-7-1 Sakamoto, Nagasaki 852-8501, Japan; mrmt.shmp@gmail.com; 4 Department of Nephrology, Nagasaki University Hospital, 1-7-1 Sakamoto, Nagasaki 852-8501, Japan; tnishino@nagasaki-u.ac.jp; 5 Health Center, Nagasaki University, Nagasaki 852-8521, Japan

In our published publication [1], there was an error regarding the affiliations of Haruka Arimori, Norio Abiru, and Atsushi Kawakami. These authors are all affiliated with the Department of Endocrinology and Metabolism, Division of Advanced Preventive Medical Sciences, Graduate School of Biomedical Sciences, Nagasaki University.

The corrected author list is provided below: 

Haruka Arimori ^1,2^, Norio Abiru ^1,2^, Shimpei Morimoto ^3^, Tomoya Nishino ^4,5^, Atsushi Kawakami ^1,2^, Akie Kamada ^1,^* and Masakazu Kobayashi ^1^

^1^ Department of Endocrinology and Metabolism, Nagasaki University Hospital, 1-7-1 Sakamoto, Nagasaki 852-8501, Japan; jj20230362@ms.nagasaki-u.ac.jp (H.A.); abirun@nagasaki-u.ac.jp (N.A.); atsushik@nagasaki-u.ac.jp (A.K.); masakazu-f328@nagasaki-u.ac.jp (M.K.)

^2^ Department of Endocrinology and Metabolism, Division of Advanced Preventive Medical Sciences, Graduate School of Biomedical Sciences, Nagasaki University, 1-7-1 Sakamoto, Nagasaki, 852-8501, Japan

^3^ Innovation Platform & Office for Precision Medicine, Nagasaki University Graduate School of Biomedical Sciences, 1-7-1 Sakamoto, Nagasaki 852-8501, Japan; mrmt.shmp@gmail.com

^4^ Department of Nephrology, Nagasaki University Hospital, 1-7-1 Sakamoto, Nagasaki 852-8501, Japan; tnishino@nagasaki-u.ac.jp

^5^ Health Center, Nagasaki University, Nagasaki 852-8521, Japan

* Correspondence: a-kamada@nagasaki-u.ac.jp

The authors state that the scientific conclusions of the original article are unaffected. This correction was approved by the Academic Editor. The original publication has also been updated.
