# Supplementary material for: Association between Lifestyle Factors and Weight Gain among University Students in Japan during COVID-19 Mild Lockdown: A Quantitative Study
Source: Healthcare (Basel). 2023 Sep 27;11(19):2630. doi: 10.3390/healthcare11192630 (PMC10572644; doi:10.3390/healthcare11192630)
Supplement: Supplementary file 1 [file healthcare-11-02630-s001.zip › Supplementary material_MI_HARCTJ-1.pdf]

Table S1. Confusion matrix and Cronbach's α coefficients of self-reported weight change in the fiscal year 2021 questionnaire and the amount of actual weight change based on student health examination data from fiscal years 2019 to 2021.

		Weight gain of ≥3 kg	Weight gain of <3 kg	No change	Weight loss of <3 kg	Weight loss of ≥3 kg
(1)	+3≦ΔBW	124	94	106	9	3
	+1≦ΔBW<+3	28	107	181	21	5
	−1≦ΔBW<+1	8	59	253	67	22
	−3≦ΔBW<−1	8	29	212	106	40
	ΔBW<−3	1	13	135	73	79
(2)	+4.5≦ΔBW	87	44	52	4	0
	+1.5≦ΔBW<+4.5	60	133	183	20	7
	−1.5≦ΔBW<+1.5	15	91	370	94	32
	−4.5≦ΔBW<−1.5	6	28	223	123	57
	ΔBW<−4.5	1	6	59	35	53

ΔBW: actual weight change based on student health examination data from fiscal years 2019 to 2021 (kg)
The Cronbach's α coefficients in ΔBW and questionnaire responses with in the measurement results stratified into five groups using criteria (1) and (2) were 0.69 and 0.71, respectively.
The sensitivity and specificity of ΔBW stratified into five groups and questionnaire responses for weight gain of ≥3 kg and ΔBW of ≥4.5 kg were 0.47 and 0.95, respectively, whereas for ΔBW of ≥3 kg, the values were 0.37 and 0.97, respectively. In the corresponding ΔBW for other groups, sensitivity was 0.26–0.62 and specificity was 0.54–0.95.

Table S2. Binary logistic regression analysis for the lifestyle factors associated with a weight gain of >3 kg during mild lockdown of the four factors with the highest OR in the Fisher's exact test in male students.

Lifestyle factors	Category	Units	Estimate of log odds ratio	95% CI	p value	
# Frequency of dining out and sleep duration						
Frequency of dining out	<1	time/week	Reference			
Frequency of dining out	1	time/week	0.03	(−0.40 to 0.44)	0.88	
Frequency of dining out	2–3	times/week	0.16	(−0.30 to 0.59)	0.48	
**Frequency of dining out**	**≥4**	**times/week**	**0.68**	**(0.17 to 1.16)**	**6.8×10^{-3}**	**
Sleep duration	6–9	h/day	Reference			
Sleep duration	<6	h/day	0.22	(−0.14 to 0.56)	0.22	
Sleep duration	≥9	h/day	0.78	(0.08 to 1.41)	0.02	*
# Frequency of dining out and gaming time						
Frequency of dining out	<1	time/week	Reference			
Frequency of dining out	1	time/week	0	(−0.43 to 0.41)	0.99	
Frequency of dining out	2–3	times/week	0.14	(−0.32 to 0.57)	0.55	
**Frequency of dining out**	**≥4**	**times/week**	**0.66**	**(0.15 to 1.14)**	**8.7×10^{-3}**	**
Gaming time	0	h/day	Reference			
Gaming time	<2	h/day	0.12	(−0.32 to 0.57)	0.61	
Gaming time	2–4	h/day	0.12	(−0.33 to 0.58)	0.60	
**Gaming time**	**≥4**	**h/day**	**0.82**	**(0.30 to 1.35)**	**2.0×10^{-3}**	**
# Frequency of dining out and internet surfing time						
Frequency of dining out	<1	time/week	Reference			

Frequency of dining out	1	time/week	0.01	(−0.43 to 0.42)	0.98	
Frequency of dining out	2–3	times/week	0.15	(−0.31 to 0.58)	0.52	
**Frequency of dining out**	**≥4**	**times/week**	**0.62**	**(0.11 to 1.11)**	**1.4×10^{-2}**	*
Internet surfing time	0	h/day	Reference			
Internet surfing time	<2	h/day	0.1	(−0.85 to 1.33)	0.85	
Internet surfing time	2–4	h/day	0.18	(−0.75 to 1.40)	0.73	
Internet surfing time	≥4	h/day	1.04	(0.10 to 2.27)	5.3×10^{-2}	
# Sleep duration and gaming time						
Sleep duration	6–9	h/day	Reference			
Sleep duration	<6	h/day	0.2	(−0.16 to 0.54)	0.27	
Sleep duration	>9	h/day	0.64	(−0.08 to 1.28)	0.06	
Gaming time	0	h/day	Reference			
Gaming time	<2	h/day	0.13	(−0.31 to 0.58)	0.58	
Gaming time	2–4	h/day	0.11	(−0.34 to 0.57)	0.63	
**Gaming time**	**≥4**	**h/day**	**0.78**	**(0.25 to 1.30)**	**3.9×10^{-3}**	**
# Sleep duration and internet surfing time						
Sleep duration	6–9	h/day	Reference			
Sleep duration	<6	h/day	0.2	(−0.16 to 0.54)	0.27	
Sleep duration	≥9	h/day	0.64	(−0.07 to 1.28)	0.06	
Internet surfing time	0	h/day	Reference			
Internet surfing time	<2	h/day	0.17	(−0.79 to 1.40)	0.76	
Internet surfing time	2–4	h/day	0.24	(−0.69 to 1.46)	0.65	
Internet surfing time	**≥4**	**h/day**	**1.09**	**(0.14 to 2.31)**	**0.04**	*

Gaming time and internet surfing time

Gaming time	0	h/day	Reference		
Gaming time	<2	h/day	0.18	(−0.27 to 0.66)	0.43
Gaming time	2–4	h/day	0.15	(−0.32 to 0.62)	0.54
Gaming time	≥4	h/day	0.52	(−0.03 to 1.07)	0.06
Internet surfing time	0	h/day	Reference		
Internet surfing time	<2	h/day	0.01	(−0.99 to 1.26)	0.99
Internet surfing time	2–4	h/day	0.09	(−0.88 to 1.34)	0.86
Internet surfing time	≥4	h/day	0.88	(−0.11 to 2.13)	0.11

*$p < 0.05$; **$p < 0.01$; ***$p < 0.01$. We conducted binary logistic regression analysis of the four factors with the highest OR in the Fisher's exact test. These top factors included frequency of dining out of ≥4 times/week, sleep duration of ≥9 h, gaming time of ≥4 h, and internet surfing time of ≥4 h.

Table S3. Binary logistic regression analysis for the lifestyle factors associated with a weight gain of >3 kg during mild lockdown of the four factors with the highest OR in the Fisher's exact test in female students.

Lifestyle factors	Category	Units	Estimate of log odds ratio	95% CI	p value	
# Frequency of dining out and smoking status						
Frequency of dining out	<1	time/week	Reference			
Frequency of dining out	1	time/week	0.04	(−0.51 to 0.55)	0.87	
Frequency of dining out	2–3	times/week	0.47	(−0.29 to 1.13)	0.19	
Frequency of dining out	≥4	times/week	0.76	(−0.34 to 1.66)	0.13	
Smoking status	None		Reference			
Smoking status	Ex		−0.74	(−4.10 to 2.62)	0.63	
Smoking status	Occasion		1.12	(−2.40 to 4.70)	0.49	
Smoking status	Daily		−0.67	(−2.46 to 2.27)	0.54	
# Frequency of dining out and time spent at home						
Frequency of dining out	<1	time/week	Reference			
Frequency of dining out	1	time/week	0.08	(−0.48 to 0.58)	0.78	
Frequency of dining out	2–3	times/week	0.5	(−0.26 to 1.16)	0.16	
Frequency of dining out	≥4	times/week	0.69	(−0.41 to 1.59)	0.17	
Time spent at home	<8	h/day	Reference			
Time spent at home	8–12	h/day	0.51	(−0.10 to 1.13)	0.11	
**Time spent at home**	**≥12**	**h/day**	**0.67**	**(0.16 to 1.22)**	**0.01**	*
# Frequencies of dining out and social club activities						
Frequency of dining out	<1	time/week	Reference			

Frequency of dining out	1	time/week	0.04	(−0.51 to 0.54)	0.88	
Frequency of dining out	2–3	times/week	0.46	(−0.30 to 1.11)	0.20	
Frequency of dining out	≥4	times/week	0.75	(−0.35 to 1.65)	0.13	
Frequency of social club activities	<1	day/week	Reference			
Frequency of social club activities	2–3	days/week	−0.01	(−0.61 to 0.65)	0.97	
Frequency of social club activities	≥4	days/week	0	(−0.59 to 0.65)	1.00	
# Smoking status and time spent at home						
Smoking status	None		Reference			
Smoking status	Ex		−0.64	(−4.00 to 2.71)	0.67	
Smoking status	Occasion		1.15	(−2.38 to 4.75)	0.48	
Smoking status	Daily		−0.59	(−2.38 to 2.36)	0.59	
Time spent at home	<8	h/day	Reference			
Time spent at home	8–12	h/day	0.5	(−0.11 to 1.13)	0.11	
**Time spent at home**	**≥12**	**h/day**	**0.67**	**(0.16 to 1.22)**	**0.01**	*
# Smoking status and frequency of social club activities						
Smoking status	None		Reference			
Smoking status	Ex		−0.61	(−3.96 to 2.74)	0.69	
Smoking status	Occasion		1.1	(−2.42 to 4.68)	0.50	
Smoking status	Daily		−0.63	(−2.41 to 2.32)	0.57	
Frequency of social club activities	<1	day/week	Reference			
Frequency of social club activities	2–3	days/week	0	(−0.60 to 0.66)	1.00	
Frequency of social club activities	≥4	days/week	0	(−0.59 to 0.65)	1.00	
# Time spent at home and frequency of social club activities						
Time spent at home	<8	h/day	Reference			

Time spent at home	8–12	h/day	0.5	(−0.11 to 1.13)	0.11	
**Time spent at home**	**≥12**	**h/day**	**0.68**	**(0.17 to 1.23)**	**0.01**	*
Frequency of social club activities	<1	day/week	Reference			
Frequency of social club activities	2–3	days/week	−0.05	(−0.66 to 0.61)	0.87	
Frequency of social club activities	≥4	days/week	0.01	(−0.58 to 0.66)	0.98	

*p < 0.05; **p < 0.01; ***p < 0.01. Non: nondrinker or nonsmoker. Ex: ex-drinker or ex-smoker. Occasion: occasional smoker. Daily: daily smoker. We conducted binary logistic regression analysis of the four factors with the highest OR in Fisher's exact test. These factors included frequency of dining out of ≥4 times/week, occasional smoking, time spent at home of ≥12 h, frequency of social club activities of ≥4 times/week.
