# Supplementary figures and images for: Association between Lifestyle Factors and Weight Gain among University Students in Japan during COVID-19 Mild Lockdown: A Quantitative Study
Source: Healthcare (Basel). 2023 Sep 27;11(19):2630. doi: 10.3390/healthcare11192630 (PMC10572644; doi:10.3390/healthcare11192630)

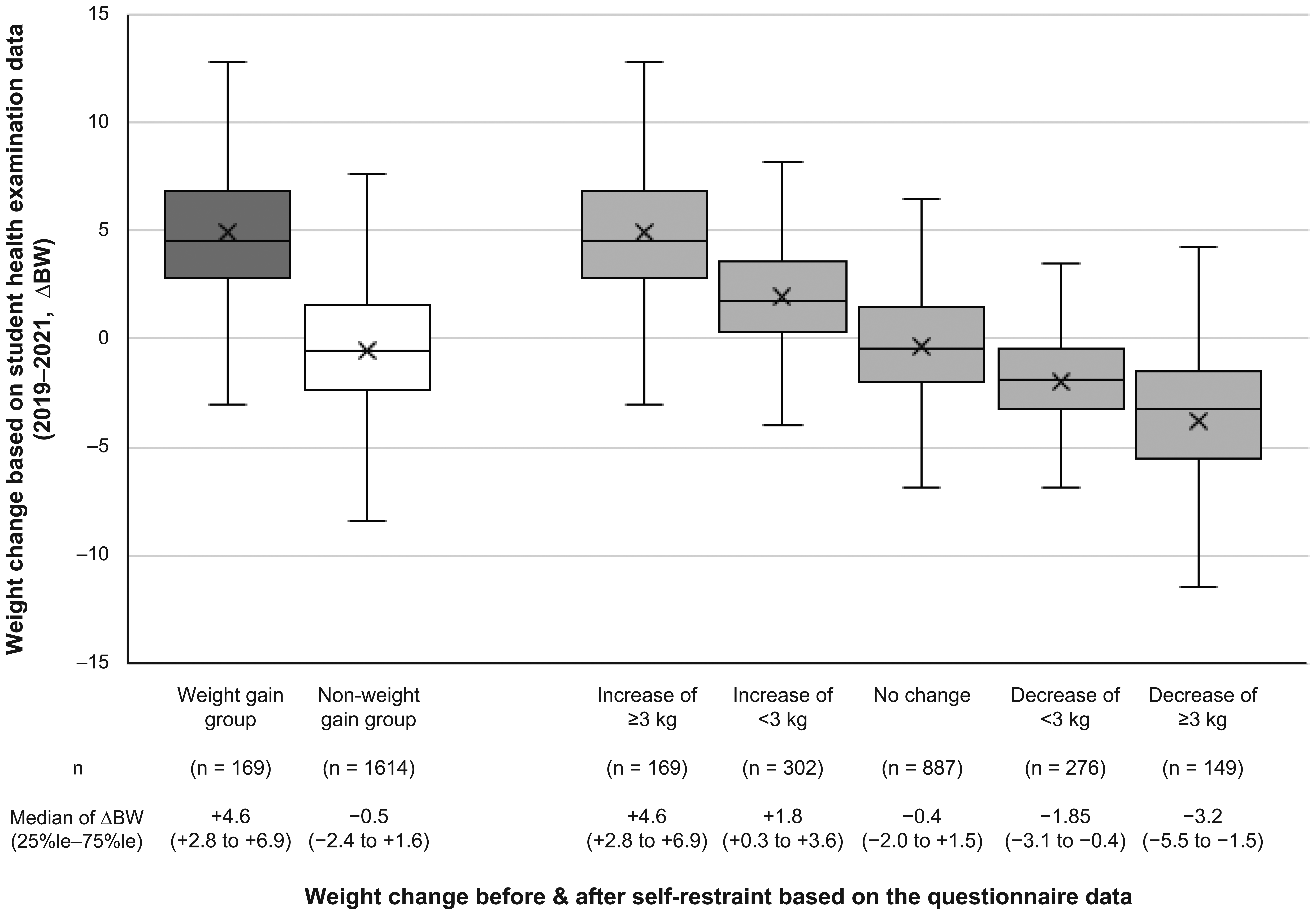

Supplement: Supplementary file 1 [file healthcare-11-02630-s001.zip › Figure_S1.tif]
